# Non-Clinical Models for Neurodegenerative Diseases: Therapeutic Approach and Drug Validation in Animal Models

**DOI:** 10.3390/bs7040082

**Published:** 2017-12-07

**Authors:** Caridad Ivette Fernandez, Jessica López, Lázara Martinez

**Affiliations:** 1 Basic Division, International Center of Neurological Restoration (CIREN), Havana 11300, Cuba; 2 Medical Surgical Research Center (CIMEQ), Faculty of Medical Sciences Victoria de Giron, Havana 11300, Cuba; jessie40997@gmail.com; 3 Cuban Society of Laboratory Animal Science (SCCAL), Veterinary Council Presidency, Havana 10400, Cuba; lazaramartinez@infomed.sld.cu

**Keywords:** animal model, behavior, neurodegenerative disease, neuroprotection, Ciren

## Abstract

In 2016, 19.8% of the Cuban population was aged 60 or over. As a result, age-associated degenerative diseases and other diseases have become priority targets from a prophylactic, diagnostic and therapeutic perspective. As a result, the Cuban biomedical scientific community has addressed its basic, preclinical and epidemiological research in order to rise up to the challenge. A firm step in this direction has been the international congress “State of the art in non-clinical models for neurodegenerative diseases” which has brought together preclinical and clinical researchers, technicians and regulatory staff members from different countries to review the state of the art in neurodegenerations, find unifying ideas, objectives and collaborations or partnership. The objective is to expose the perspectives of new biotechnological products from Cuba and other countries from the diagnostic, therapeutic and neuroprotective point of view. It is crucial, therefore, that the irreplaceable role of laboratory animals in achieving these objectives is understood but they must be used in rational, adequate and ethical manner. We expose the current development trends in this field, being of common interest to the work directed to the search for potential drugs, diagnostic tools and the promotion of changes in lifestyle as a preventive projection.

## 1. Introduction

What was the rationale for the Cuban Society of Laboratory Animal Science organizing the First International Congress on non-clinical models for neurodegenerative diseases?

The Cuban Statistical Yearbook of Health 2016 shows that in three categories of causes of death, the rate of mortality from non-transmissible chronic diseases is the highest, with 731.4 deaths per 100,000 inhabitants. In the top ten, heart disease occupiesthe first place with a rate of 217.7 per 100,000 inhabitants, followed by death from malignant tumors at 216.3. These top two account for 49.1% of the total of the deaths in 2016. According to death records, among the 35 leading causes of death for both sexes, dementia and Alzheimer’s disease (AD) placed 6th, while cerebrovascular disease (CVD), diabetes and Parkinson’s disease (PD) were third, tenth and seventeen places, respectively [1].

When tabulated, the average population skews towards the aged. This is also observed in developed countries that have largest proportion of elderly population. Other demographic indicators, including birth indicators, total fertility rate (children per woman), gross reproduction rate (daughters per woman), percentage of the adult population and the life expectancy at birth corroborate this phenomenon. This is a result of improved public health standards, with free medical services, wide health promotion and disease-prevention programs, early detection and/or vaccination schedules, as well as a solid national program of diagnostic, therapeutic and pharmaceutical biotechnology. This is built around national needs, and eliminates the need for medical imports and dealing with the restrictions associated with foreign regulations imposed by US legislation in the international market and offers assistance to other countries.

The life expectancy at birth for Cubans is 78.45 years. In 2016, 19.8% of the Cuban population was aged 60 years or more. As a result, age-associated degenerative diseases and other diseases with well-defined risk factors such as diabetes mellitus and hypertension (cerebrovascular disease) have become priority targets from a prophylactic, diagnostic and therapeutic perspective. As a result, the biomedical scientific community has adjusted its basic, preclinical and epidemiological research to rise up to the challenge. This change in demography has modified the current and future strategic projections of the Public Health Ministry, the Academy of Science, the Cuban medical-pharmaceutical and biotechnology industry as well as the extensive network of collaboration between medical, technical and social universities, health institutes by specialties (tertiary level), hospitals and the primary level of community care that National programs are operating directed to the search of potential drugs, diagnostic tools and the promotion of changes in lifestyle as a preventive projection [1,2].

The objective is to challenge all levels of biomedical and preclinical research to achieve long-term diagnostic, therapeutic and prophylactic products. It is crucial, therefore, that the irreplaceable role of laboratory animals in achieving these objectives is understood. However, they must be used in a rational, adequate and ethical manner.

This congress has brought together preclinical and clinical researchers, technicians and regulatory staff members from different countries and with different research interests and expertise, to review the state of the art in neurodegenerations, find unifying ideas, objectives and collaborations or partnerships. Participants represent experts from Cuba, Brazil, Colombia, France, Germany, Italy, Japan, Mexico, Spain, Turkey, and USA in the field of experimental animals, neurodegenerative diseases and associated biomodels, ranging from the molecular level to the systemic/functional level.

Cuba and particularly the Cuban Society for Laboratory Animal Sciences (SCCAL) acknowledges and appreciates the presence, support and active participation of all delegates as well as to their scientific and human contribution to the development of the congress, particularly to the International Council of Laboratory Animal Science (ICLAS) and the American Association of Laboratory Animal Science (AALAS) that in the presence of its officials (Dr. Cynthia Pekow, General Secretary of the ICLAS, Dr. Ekaterina Rivera, co-chair of the American Regional Committee at ICLAS and President of the Brazilian Society of Laboratory Animal Science (SBCAL) and Dr. Ann Turner, Executive Director AALAS), became part of our family and vice versa. From this perspective article we focused the attention in the “Non-clinical models for neurodegenerative diseases: therapeutic approach and drug validation in animal models” congress session. Those aspects concerning other sessions will be summarized through other perspective articles in this special issue.

## 2. Non-Clinical Models for Neurodegenerative Diseases: Therapeutic Approach and Drug Validation in Animal Models

### 2.1. Animal Models in the Restorative Neurology Context

The inaugural address summarized the mandate of the International Neurological Restoration Center (Ciren, Havana, Cuba), a nascent Cuban institution that amalgamates academics with national medical services and also provides health tourism services to patients afflicted with neurological disorders from more than twelve countries. Dr. Caridad I. Fernandez (Ciren), who moderated this session, explained how the human, medical and technological resources are organized to support a multidisciplinary and multifactorial scientific strategy that brings together basic, preclinical and clinical divisions to facilitate a seamless translational process. This effort includes assimilation of technology and implementation of discoveries from the basic sciences to develop new clinical diagnostic tests, therapies and prophylactics based on animal models of neurodegenerative diseases (NDDs).

The nexus of understanding the nervous system and novel therapeutic interventions including nutrition and daily life activities, pharmacological modulation, direct or indirect sensory, motor and cognitive stimulation; functional correction of neural circuits using functional surgery techniques by minimal access with neuroimaging assessment/support, cell replacement therapy by stem cells or stimulation of endogenous neurogenesis by training, cytokines, transcranial magnetic stimulation, have been used to target reorganization of synaptic and neural network connections, remodeling of the brain parenchyma and its systemic connections to promote restoration of higher-order cognitive, behavioral, and sensorimotor functions. The functional clinical neurorestorative program assimilates basic, preclinical and clinical data based on neuroregeneration, neurorepair, neuroplasticity, neuroprotection, neuromodulation, neurorehabilitation to achieve Restorative Neurology. Dr. Fernandez concluded that the selection of an appropriate animal model and design of the experiments and clinical trials will be the decisive factor in promoting more efficacious therapeutic interventions and solve contradictions between preclinical and clinical trials and move the field forward.

### 2.2. Therapeutic Animal Models of NNDs

Dr. Pamela Maher (The Salk Institute for Biological Studies, La Jolla, CA, USA) pointed out that there are no drugs that halt the progression of any age-associated neurodegenerative disease. This is likely due to the failure of drug developers to recognize that while there are mutations that predispose individuals to disease as they get older, the vast majority of neurodegenerative diseases arise from a confluence of multiple, toxic insults. Thus, it is unlikely that the current reductionist approach is going to yield useful drugs for these conditions. She emphasized that the identification of multi-target lead compounds is needed and their selection should be based upon a requirement for their efficacy in phenotypic screening assays that reflect the biology of the aging brain. This approach to neurodegenerative disease drug discovery is likely to produce safe and effective drugs. She noted the roles of plant secondary metabolites (natural products) are the ideal source of lead compounds because they have evolved to interact at low affinity with multiple enzymes. Indeed, the chemical scaffolds of the majority of drugs in the clinic are based upon those of natural products. Her laboratory has completed a series of proof of principle experiments demonstrating that some polyphenolic natural products are exceptionally effective in halting disease progression in a wide variety of animal models of age-associated neurodegenerative disease and ischemia, and that the potency and medicinal chemical properties of these compounds can be dramatically improved without losing their multi-target activities. In conclusion, she pointed out that the single-targethigh-affinity drug approach to neurodegenerative disease is unlikely to identify compounds that halt disease progression. Drug discovery paradigms based upon phenotypic screening are more likely to succeed.

A significant group of Cuban clinical/preclinical investigators based their presentations on NDDs for which there iscurrently no effective treatment available, their major impact on quality of life, as well as associated chronic diseases with high socioeconomic costs for both, state and family. These researchers justified their presence in a meeting of “laboratory animals” because these disorders will become even more important for our society in the future and there is a need for the development of new, adequate treatment options and basic and preclinical studies play a decisive role on this aspect. On the other hand, experts such as George Perry (University of Texas, San Antonio, TX, USA), Dr. Joseph H. Neale (Professor Emeritus, Georgetown University, Washington, WA, USA), Lara Ordóñez-Gutiérrez and Francisco Wandosell (Center for Molecular Biology “Severo Ochoa”-CSIC-UAM-, Madrid, Spain) reviewed evidence and results for the existence of multiple endo/exogenous factors that affect the clinical course of NDDs such as the mitochondria role in oxidative stress, neurotransmitter-mediated excitotoxicity, proteinopathies and brain metabolism, respectively. Their concluding remarks emphasized the multifaceted role of these factors so that they should be taken into account as potential therapeutic targets, considering that today there are few drugs approved for the symptomatic treatment of these diseases, so the search for interventions that cause changes lasting in the clinical evolution of a NDD and therefore modify those events that lead to death and neuronal degeneration (named as disease modifying therapies), constitutes a high-priority element, it was concluded. 

At poster session, a very controversial aspect was the fact that rather than replacing the lost neurons, the neurorestoration advocates the protection of the different affected circuits and the dynamic recovery of the adult neuronal and neural network homeostasis and plasticity. These processes are based on a continuum of cellular stress responses and systems adaptations during brain aging, where pro- and anti-inflammatory events will be decisive for neuronal death or survival, particularly modulated by astroglial activity, which was emphasized by Dr. Tomás R. Guilarte (Florida International University, Miami, FL, USA), who described the validation of the Translocator Protein 18 kDa (TSPO) as a biomarker of brain injury and inflammation in different models of neurodegeneration and its clinical use in human neurodegenerative disorders. He also stressed the importance of biomarkers of neuroinflammation and brain injury to reveal the effects of toxicants in the CNS, and subsequently developing strategies to mitigate neurological effects as well as the important value of searching, validation and application of new specific biomarkers available in the correlation of functional and structural findings for diagnostic and therapy. On the other hand, Gianna Palmiere (Institute of Biosciences and Bioresources (IBBR)-Napoli, Italy) pointed out the role of the collection of multiple biomarkers to support diagnosis and disease modification induced by therapeutic approach. She illustrated the importance of newly identified peripheral biomarker systems, demonstrated for the first time the existence of a relationship between the blood APEH-proteasome levels and AD, laying the foundations for a possible use of this enzyme as a new biomarker and therapeutic-target in neurodegenerative diseases.

Fabrice Leclerc (Université Paris Saclay, Université Paris Sud, Orsay, France) provided a “magical touch of science fiction” with his presentation on RNA bioinformatics. Leclerc et al. reported the combined experimental and computational approaches in transcriptomics and interactomics of RNA-binding Proteins (RBPs) to develop computational approaches to characterize the RBPs interaction networks, their contributions to CNS homeostasis as well as to identify potential new targets. Dr. Leclerc proposed an in silico design of specific RNA ligands (fragment-based approach) astherapeutic strategy to restore the RBPs homeodynamics in neurodegenerative disorders.

### 2.3. Animal Models of Neuro-Disabilities: Different Strategies to Define NeurorestorAtive Therapies

Dr. Maurice Tangui from the University of Montpelier (France) provided an overview on the sigma-1 receptor (S-1R) agonists because of their cytoprotective action in different neurodegenerative pathologies including Alzheimer’s disease (AD), Parkinson’s disease (PD), Huntington’s disease (HD), amyotrophic lateral sclerosis (ALS), or stroke. Tangui concluded that results from pharmacological and genetic animals models of neurodegenerative conditions indicate that the S1R and its agonists constitute an effective endogenous neuroprotection system and a possible disease-modifying agents respectively. 

An important part of the session included an expert panel to show basic/preclinical studies addressed to demonstrate the neuroprotective potential of a variant of the human recombinant erythropoietin (rhEPO) with low sialic acid content (NeuroEPO) in several animal models. Dissertations included relevant specialists from the NeuroEPO producer/developer center (CIM; Center of Molecular Immunology, Cuba) and different profiles evaluators (Dr. Daniel Amaro, Dr. Teresita Rodriguez, Dr. Iliana Sosa). More than twelve communications showed the neurological restoration induced by NeuroEPO treatment in non-transgenic, transgenic and natural models of Alzheimer’s disease. A significant amount of histological findings, behavioral, biochemical and pharmacological facts were discussed, supporting the advantages of NeuroEPO by nasal route as to neuroprotective therapy, particularly in the field of cognitive domains affected by neurodegenerative conditions or brain vascular lesions. Unlike other molecules of EPO, which have no capability for erythropoiesis yet retain their neuroprotective capacity, the NeuroEPO has demonstrated no side effects associated either to the route or the product by itself, at the CNS level or systematically. As support to the introduction of this new formulation of the rhEPO studies were shown in fimbria-fornix injury models in rats, in naturally aged macaques with cognitive deterioration and in a PD clinical trial as shown by the Ciren, Cetex and Cenpalab specialists, showing good tolerance and effects positive in some cognitive functions, sustaining the assumption that EPO can act not only as a neuroprotective substance, but is also able to modulate transient neural plasticity mechanisms and therefore to promote the recovery of nerve function after an established chronic brain lesion. Evaluation of NeuroEPO in a transgenic mouse with the SCA-2 ataxia gene exhibits the neuroprotective potential of this drug, similar to above cited studies in other models.

From Barcelona Univ Dr. R. Rama gave the results of the study to know the cellular and molecular mechanisms by which NeuroEPO exerts its neuroprotective action on stroke, using an in vitro model. The results confirm that NeuroEPO exerts a restorative effect on neuronal damage induced by excitotoxicity. Without discarding other mechanisms, NeuroEPO improves the antioxidant activity in the neuron and protects it from oxidative stress. Considering that the new CIM formulation of NeuroEPO constitutes an additional benefit over other variants of EPO for the treatment of neurodegenerative conditions, the corresponding Cuban regulatory entity announced the approval to initiate clinical trials in the initial stages of AD and PD. Table 1 summarize biological products whose therapeutic potential were presented by different Cuban institutions and collaborators labs in Europe. 

The most numerous and relevant results from young researchers from Latin American research groups came from the Research Group Experimental Models for Zoohumans Sciences, University of Tolima, Ibagué Colombia, who evaluated promising anti-parkinsonian effects of *Mucuna pruriens*, a phytodrug, in an unconventional model of dyskinesia in rats evaluated in addition in a novel system. 

The design of new tools for the simultaneous quantitative evaluation of different behavioral parameters in models of primary motor neurodegenerative diseases was addressed by Dr. Liliana Francis (Tolima University, Colombia). In the framework of a practical session she demonstrated the Turner box utility to evaluate the cognitive-motor and functional asymmetry impairments. This new tool will become useful for the integral evaluation of these behavioral parameters.

### 2.4. Animal Models of Neuro-Disabilities: The Value of Animal Care and Welfare for Its Use in Neuroscience

Animal models have become an indispensable tool, both to increase knowledge about the etiology and pathology of NDDs and, consequently, for the development of new therapeutic approaches. However, reality today shows that most of the drugs that have been developed for the treatment of these degenerative conditions with promising results obtained in preclinical studies have not been successfully reproduced in the clinical studies. One of the key points of this divergenceis the choosing of the suitable animal model during the preclinical phase of research. This theme was widely discussed in the state of the art in the non-clinical studies for AD, using animal models, referring torelevant aspects, the symptomatic value of the naturally aged, transgenic mouse BALB/c, (Dr. Alain Morejon and Dr. YenelaGarcía, Biocen, Cuba) and the APOE-KO mice modeling the age-related neurodegenerations (Dr. Dasha Fuentes, Cetex-Cenpalab, Cuba). This sessionhighlighted the relevant value of aged non-human primates (NHP) with “alzheimerian” and “parkinsonism” manifestations as models of AD and PD (Dr. Caridad I. Fernandez, Ciren). Primatologists withinCuban research centers (CIGB and CNC) offered longitudinal behavioral, hematological and biochemical profiles of different Macaca species. Dr. Rafael Martinez and Dr. Pedro Puentes made particular emphasis on monkeys with obesity-related metabolic syndrome as obesity/diabetes animal models as well as their value in the context of neurodegenerative diseases. Dr. Lazara Martinez, President of the Cuban Society of Laboratory Animal Science (SCCAL, acronym from Spanish) stressed that the NHP use will always require the maximum supervision of the ethical and bioethical regulations in terms of animal use and welfare. 

Concluding the work session, the studies and practical activities from Tecniplast SPA (Eng. A. Peruffo, Italy) were particularly attractive, showing how the original need to have just “container” for rodents has evolved over time to meet the need of a range of practical, scientific requirements: health status, bio-containment or bio-exclusion, separation of small groups of animals, space saving, ergonomics, practicality of use and, last but not least, animal and human welfare. Compliance with these criteria is essential in order to avoid undesirable variables and achieve reliable scientific outcomes. However, the high price of these systems prevents their normative standardization in all laboratories and bioterios. In an effort to translate the environmental concerns from the cage to the human house and community, there was consensus that a multifactorial therapy seems to be the best approach in neurological diseases in which the lifestyle and activity-promoting environments play a significant role, according to the results shown with environmental enrichment in rodents, NHP and humans by Dr. P. Puentes and Dr. Alain Y. Garcia (Ciren).

Studies with in vitro systems were also subject of discussion, such as an alternative to animal models. Culture collection of microorganisms and other microbiological materials are essential to biotechnology development as explained by Dr. Barbara Gonzalez (CIGB, Cuba). She reviewed the use of experimental animals as biomodels with limitations because regulatory ethical precepts regarding cellular and microbial systems are highly demanded as a successful alternative.

The value of adequate management of good laboratory practice (GLP) systems and organizational structure that allow traceability of all samples from regulated non-clinical studies was stressed by Dr. Gonzalez from the CIGB working group. They emphasized the practical value of developed programs for compliance guidelines and effective training of professional and technical personnel based on National Regulatory Body and International Standards. Implementation of these regulations is imperative but it has specific challenges in the context of each country, she concluded.

### 2.5. Animal Care and Use Programs: Societies and Organizations of Laboratory Animal Science

Under the auspice of ICLAS, AALAS and SCCAL, represented by Dr. Cynthia Pekow, General Secretary of ICLAS, Ann Turner, Executive Director AALAS and Dr. Lazara Martinez, President of the SCCAL, the laboratory animal science was reviewed addressing the main current development conflicts as well as the role of scientific and professional associations/societies for animal care and use. Evidence of the implementation of these programs and normative and educational materials was shown by CIGB specialists for the introduction of positive reinforcement training methods in monkeys. It also constitutes a refinement of animal management methods that help improve animal welfare, veterinary care, and the value of animals as research subjects, relevant issues particularly in animals’ uses for behavioral studies. This special issue emphasized not only the need for appropriate animal models to increase our understanding and knowledge, but also for the final clinical application of regenerative medicine-based products.

## Figures and Tables

**Table 1:** Potential therapeutic compounds presented and discussed during the work session.

Potential Drugs	Evaluated Function	Model(s)	Group/Laboratory *
EPO	Neuroprotection, Motor and cognitive functions, antioxidant activity	Fimbria-fornix lesion;cognitively impaired aged monkeys Clinical trial Phase I in PD patients (closed with results). New PD clinical trial is open	CIREN, Cuba
G-CSF	Neuroprotection, Motor and cognitive functions, antioxidant activity	Cognitively impaired aged monkeys	CIREN, Cuba
Neuro-Epo	Antioxidant activity, neuroprotection	Neuronal primary cultures	CIM; Cetex (Cuba) Barcelona Univ., Spain
Sigma-1 receptor (S1R) agonists	Neuroprotection	Pharmacological and genetic AD animals models	INSERM Montpellier Univ., France
Compvit-B^®^	Neurogenesis	Fimbria-fornix lesion; cyatic nerve lesion in rats; Spino-cerebellousathaxiatype 2 disease	CNC, Cuba
Phytodrug (*Mucunapruriens*)	Motor functions	Hemi-parkinsonian rats; dyskinesia rat model	Tolima Univ., Colombia
Amylovis	Cognitive functions; amyloide clearance	Pharmacological model of amyloidosis in aged mice	CNC, Cuba
Conditioned media/secretomefrom MBMSC	Neuroprotection, Motor and cognitive functions, antioxidant activity	Aged, cognitivelyimpaired rats	CIREN, Cuba

* All cited acronyms are from Spanish. CIGB: Center of Genetic Engineering and Biotechnology; Cetex: Ctr of Experimental Toxicology; Cenpalab: National Center for laboratory animal breeding; CIM: Ctr of Molecular Immunology; Ciren: Intl Ctr Neurological Restoration; CNC: Cuban Neuroscience Center. MBMSC: mesenchymal bone marrow stem cells.
